# Pertinence of the recent school-based nutrition interventions targeting fruit and vegetable consumption in the United States:a systematic review

**DOI:** 10.15171/hpp.2016.01

**Published:** 2016-03-31

**Authors:** Christopher R. Aloia, Taylor A. Shockey, Vinayak K. Nahar, Kathy B. Knight

**Affiliations:** ^1^Department of Nutrition and Hospitality Management, University of Mississippi, 108 Lenoir Hall, PO Box 1848, University, MS 38677, USA; ^2^Department of Health, Physical Education, and Exercise Science, Lincoln Memorial University, Mary Mars, 6965 Cumberland Gap Parkway, Harrogate, TN 37752, USA; ^3^Department of Health, Exercise Science & Recreation Management, The University of Mississippi, 215 Turner Center, PO Box 1848, University, MS 38677, USA

**Keywords:** Nutrition intervention, Fruit and vegetable consumption, School, Systematic Review

## Abstract

**Background:** Schools are the major locations for implementing children’s dietary behavior related educational or interventional programs. Recently, there has been an increase in school-based nutrition interventions. The objective of this systematic review was to overview the evidence for the effectiveness of school-based nutrition intervention on fruit and vegetable consumption.

**Methods:** PubMed was used to search for articles on school-based nutrition interventions that measured students’ fruit and vegetable consumption. Our search yielded 238 articles.The article was included if published in a peer-reviewed journal, written in English language,administered in the United States, and conducted among a population-based sample of children in Kindergarten through eighth grade. A total of 14 publications met the inclusion criteria.

**Results**: Eight articles successfully showed the positive effect on increasing fruit and or vegetable consumption while the other six did not. Several factors, including (but not limited to) intervention duration, type of theory used, style of intervention leadership, and positively affecting antecedents of fruit and vegetable consumption were compared; however, no dominant factor was found to be shared among the studies with significant findings. Given that the criteria for selection were high, the lack of consistency between interventions and positive outcomes was surprising.

**Conclusion:** With high levels of scrutiny and budget constraints on school nutrition, it is imperative that more research be conducted to identify the effective intervention components.

## Introduction

Childhood obesity rates have continued to increase in the United States leading to an explosion of food-based obesity prevention interventions in US public schools over the last decade.^1-4^ Because the US government mandates school attendance for children and adolescents, school-based nutrition programs have become increasingly prevalent to prevent this trend.^5,6^ One of the main goals of school-based nutrition programs is to increase fruit and vegetable consumption in school-aged children.^5-9^ These programs and interventions are considered as the top priority for the US government.^7,8^

As many national studies have confirmed, childhood obesity is a major issue with dire consequences on public health. Research suggests that effective intervention strategies at public school levels are required in order to control childhood obesity.^9,10^ The key question that needs to be asked, however, is – how can researchers actually change behaviors in children? Researchers have recruited parents, teachers, and celebrities to help in controlling the American obesity crisis.^7,11^ Many prevention efforts include interventions focused on increasing physical activity; however, others attempt to get children to eat his or her veggies – but what exactly does that mean? Fruits and vegetables have played a key role in a healthy diet and preventing the risk of heart disease for many years.^12-14^ Research published in projects of the American Heart Association by 2030, 116 million Americans will be diagnosed with some forms of cardiovascular disease.^15^ The US Department of Agriculture and Health and Human Services recommends consuming two-and-a-half cups of vegetables and fruits per day.^15,16^ The Union of Concerned Scientists calculated that if each citizen followed the federal recommendations, 127 261 lives would be saved annually.^17^

Previous systematic reviews that have focused on increasing fruit and vegetable consumption and/or improving antecedents of fruit and vegetable consumption have reported contradictory results.^18-23^ For example, some reviews promoted particular components based on their effectiveness of improving antecedents of fruit and vegetable consumption, such as preferences or knowledge, but these components did not demonstrate an increase in consumption. Moreover, some of these reviews included studies without control groups.^18-23^ Many of these reviews promote the idea that if each citizen increases his or her fruit and vegetable consumption, the Unites States would save $11 trillion in health care costs from heart disease.^17^ Claims of this magnitude demand an in-depth analysis of the available literature in order to understand the feasibility of such a position. When attempting to increase the fruit and vegetable consumption of public school students, it is important that health researchers and school health practitioners understand the quality and position of the evidence that is being used as support.

Given the magnitude of these claims, it is important to conduct a systematic review of recently published literature to assess the effectiveness of increasing fruit and vegetable consumption through school-based nutrition interventions. The review will then attempt to ascertain the most efficacious key components of each intervention, and will also report the overall quality of the evidence.

## Materials and Methods

### Literature search process

For this systematic review, we conducted searches (during December 2014) in PubMed (including MEDLINE) to assess primary articles published in the English language within the last decade (2004-2014). No attempt was made to assess gray literature. The keywords for this review were selected from previously published articles in the area of school-based nutrition. The following keywords were used to capture pertinent literature: “*school-based nutrition intervention,” “fruit and vegetable consumption,” “school-based childhood obesity programs,” “marketing healthy food to children,” “school based nutrition intervention,” “promoting healthy habits in school-children,”* and “*fruit and vegetable consumption in children.*”

This research was performed using the Evidence Analysis Library Manual by the Academy of Nutrition and Dietetics as a rubric for the objective analysis of the articles.^24^ All references retrieved from the database were entered in the University’s electronic file-sharing software. Microsoft Excel, 2013 was used to cross-tabulate intervention components and significant findings.

**Figure 1:**
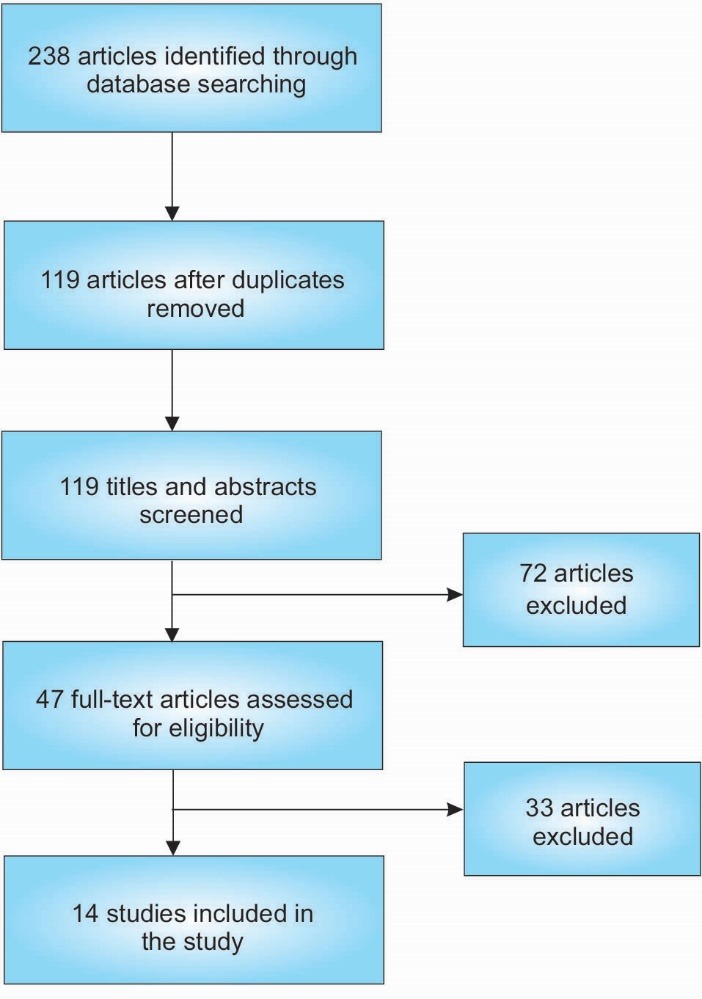
Flow chart of literature search.

After eliminating duplicate studies, titles and abstracts of all relevant articles were screened initially. Full text articles were then retrieved and reviewed based on the predefined eligibility criteria. The aforementioned literature search procedure was conducted independently by the three primary reviewers (CRA, TAS, and KBK). Any disagreements between the reviewers were resolved by discussion. Figure 1 presents the findings of our literature search.

**Table 1:** Summary of the reviewed studies

**First author (Year)**	**Study design**	**Study sample**	**Assessment method**	**Intervention**	**Findings**	**Measurements** **(F&V intake)**
Blom-Hoffman et al^25^ (2008)	Randomized at school level	297 kindergarteners and first graders	Questionnaire	5 interactive children’s books	In the experimental group fruit and vegetable consumption increased while the control group’s consumption remained stable but not significant. These results remained true for the post-intervention year 2.	Self-reported
Cohen et al^26^ (2014)	Randomized controlled school and community based	432 elementary aged children	2007 Block Food Screener	Food service component: offering whole grains daily, providing five different fruit and vegetable options weekly (with a fresh fruit or vegetable option daily, and a dark green or orange vegetable or fruit at least 3 times per week), providing beans or peas weekly, supplying 1% and nonfat milk daily, limiting ice cream sells, and encouraging a healthier a la carte portfolio Educational curriculum intervention: exposing students to the Shape Up: During - and After - School curricula, the Eat Well Keep Moving curricula, and the 5-2-1 messages	Children in the intervention groups consumed significantly more vegetables and combined fruits and vegetables than the control group. There were no significant differences between the intervention and control schools in fruit, legume, whole grain, or dairy consumption.	Self-reported; questionnaire
Hoffman et al^27^ (2010)	Longitudinal	297 kindergarteners and first graders	Questionnaire (adapted version from the Fruit and Vegetable Preference Questionnaire)	School-wide component: loudspeaker announcements providing an interesting fact about the “fruit and vegetable of the day” made by a respected adult in the school. Classroom component: 5-A-Day adventures CD-ROM used during computer class. Lunchtime component: hanging cafeteria posters reflecting the fruit and vegetable of the day and lunch aides giving verbal praise and a sticker to students eating the fruit and vegetable of the day. Family component: series of interactive children’s books assigned as homework, the 5-A-Day Kids Cookbook, and a school cookbook developed by children, parents, and teachers.	Fruit preferences were higher than vegetable preferences and preferences remained stable across time. However these preferences were not significant after adjusting for pre-intervention preferences. Children in the experimental group ate more fruits and vegetables than the control group in both years of the program. These results were statistically significant.	Self-reported; questionnaire
Parmer et al^29^ (2009)	School-garden intervention	115 2nd grade students	Survey; Questionnaire; Lunchroom Observation	Intervention #1: received nutrition education Pyramid Cafe and Health and Nutrition from the Garden for one hour. Lessons were every other week; the other two classes were assigned to the second intervention. Intervention #2: nutrition education, Pyramid Café, Health and Nutrition from the Garden 1 hour lessons every other week, and 1 hour of gardening lessons every alternating week.	Nutrition education improved fruit and vegetable knowledge and preferences in students compared to the control group. However, nutrition education and gardening improved fruit and vegetable knowledge, preferences, and vegetable consumption significantly more compared to the control group and the nutrition education intervention group. Consumption for vegetables increased for the nutrition education and gardening group but not for the nutrition education group	School-garden intervention
Prelip et al^30^ (2012)	Quasi-experimental pretest/posttest research design	399 3rd, 4th, and 5th grade students	Questionnaire	First intervention: traditional Network-LAUSD program, standardized nutrition curriculum (HOM program, the Dairy Council of California, and 5-A-Day Power Play), teacher training workshops, and parent nutrition education workshops. 2nd intervention: the traditional Network-LAUSD program and teacher training workshops.	The first intervention resulted in a significant change in knowledge, attitudes, and beliefs toward the consumption of vegetables. There were small increases in knowledge, attitudes, and beliefs toward the consumption of vegetables in the second intervention group compared to the control group. Teacher influences on students fruit and vegetables attitudes was not significant. There was no significant difference between the treatment group and control groups for F&amp;V consumption.	Questionnaire
Prelip et al^31^ (2011)	Non-randomized pre-test/post-test research design	1528 3rd, 4th, and 5th grade students	23-item Day in the Life Questionnaire, survey	Experimental schools: teachers were allowed to design their own intervention by choosing from a variety of strategies and activities. All school ended up creating their own hybrid interventions that was a combination of district strategies, local school defined strategies, and “home-made” strategies/activities created by teachers.	Interventions resulted in a significant change in teacher influences on student’s fruit and vegetable attitudes. These interventions did not have a significant effect on actual fruit and vegetable consumption. There were slight increases in fruit and vegetable consumption but not enough to be considered significant.	Survey, questionnaire
Puma et al^32^ (2013)	Quasi-experimental comparison	191 6th-8th grade students	Survey, BMI	Intervention group: INPAP intervention curriculum delivered by a resource teacher for 2 consecutive years. The curriculum was aimed to increase fruit and vegetable consumption and intensify physical activity levels by targeting simple and consistent messages and reinforcing them in multiple ways.	The intervention group did not have significantly higher intakes of fruit and vegetables compared to the control group. However, the intervention group’s nutrition-related knowledge and attitudes did increase but not their self-efficacy or behavior change.	Survey
Siega-Riz et al^33^ (2011)	Cluster-randomized design	3908 students followed from 6th grade to 8th grade	Block Kids Questionnaire	Intervention: semester themes: consuming water versus sweetened beverages, increasing physical activity and reducing sedentary behaviors, consuming high quality versus low quality foods, understanding energy balance, strength, and making choices for life. Implementation: increase healthier options for lunch and breakfast, offer at least two or three different choices for vegetables at school breakfast and lunch, modifying dessert and snack food options, eliminate beverages with added sugar and offer only 1% or skim milk, and offer at least two or three high fiber foods at lunch and breakfast.	There were significant differences between the intervention and the control groups for intakes of fruit and water. Average daily fruit consumption was 10% higher at the end of the study for the students in the intervention group compared to the control group. The intervention group also consumed 2 fluid ounces more of water than the control group at the end of the intervention. There were no significant differences between intervention and control student for mean intakes of macronutrients, fiber, grains, vegetables, legumes, sweets, sweetened beverages, and higher- or low-fat milk consumption at the end of the intervention.	Food Frequency Questionnaire
Slusser et al^34^ (2013)	Quasi-experimental pretest/posttest comparison group design	121 3rd-5th grade students	Catch Club Kids and Day in the Life Questionnaire, Previous Day Physical Activity Recall	Intervention: staff training in nutrition, child development, enhanced physical activity routines, curriculum resources, regular mentoring, and technical assistance visits. Students participated in the Catch Kids Club curriculum.	There were significant increases in nutrition knowledge and a significant decrease in junk food consumption compared to the control schools. There were no significant differences between the control and intervention groups with regard to changes in nutrition related attitudes and behaviors (eating vegetables and healthier eating choices). Intervention groups did experience a significantly greater decrease in BMI compared to the control group.	Self-reported
Spiegel et al^35^ (2006)	Randomized	1013 4th and 5th grade students	Derived survey from the CDC’s Youth Risk Behavior Survey	Intervention group: used the WAY program. The WAY program: engagement time from 20 minutes to 1 hour or more, engaged students in multidisciplinary activities in language arts, mathematics, science, and health content. Activities are sequenced to build on previous activities and students’ understandings, beliefs, and behavioral skills. Classes followed a 10-minute aerobic routine each day during class time. Seven modules included in the WAY program: an introduction to the program, teaches students how to collect, report, and analyze data about their self, focuses on physical activity and fitness, addresses nutrition and the way we eat, teaches students about their bodies, provides an orientation to genetics and family health history as a resource to examine personal health, and teaches students to learn to bring home information and skills they learned in class.	There were significant positive shifts in BMI, improved consumption of fruits and vegetables, and increased physical activity compared to the control group. Physical activity levels and changes in reported nutritional intake were notable but not significant.	Self-reported (students, teachers, and parents)
Springer et al^36^ (2012)	Quasi-experimental nonequivalent control group design	511 4th and 5th grade students	7-item scale adapted from the GEMS study, School Physical Activity and Nutrition survey, Active Kids Project, Athletic Identity and Physical Activity Questionnaire	Intervention: Marathon Kids - Children tracked the number of miles they walked or ran along with the number of fruits and vegetables they ate by coloring in their MK Mileage Log and MK Fuel Log for each quarter mile ran/walked and each fruit/vegetable consumed. Successful completion is based on walking or running 26.2 miles over a 6 month period and eating fruit or vegetables 5 times a day for 26 days or 1 month. Students were given time during school to complete their activities and had celebratory events and rewards for when they completed the intervention.	The intervention group had a higher mean time of running in the past 7 days, higher fruit and vegetable consumption, physical activity outcome expectations, but students only self-reported eating significantly more vegetables at school. Other variables, like fruit, self-efficacy and social support for the two groups did not differ significantly.	Self-reported
Struempier et al^37^ (2014)	Quasi-experimental	2477 third grade students	What’s for Lunch checklist	Intervention: Included Body Quest curriculum, iPad app education, and weekly fruit and vegetable tastings. Body Quest curriculum - 17 weekly 45 minutes classes: two weeks of pre-intervention, 13 weeks of intervention, and 2 weeks of post-intervention. Classes included trying new foods, information on food groups, balanced meals, food nutrients, healthy snacks, and extending fruit and vegetable messages to others.	There were significant increases in fruit and vegetable consumption for the intervention group compared to the control group. Most of the changes happened by class 10 for both fruits and vegetables. All fruit and vegetable predictors were significantly higher and included gender for vegetables, race for fruits and vegetables, and free/reduced lunch and fruit.	Self-reported
Wilson et al^38^ (2012)	Randomized controlled trial	1119 with a mean age of 12.7 (middle school)	Questionnaire derived from the 2005 Youth Risk Behavior Surveillance System survey and the 2005 Virginia Youth Tobacco Evaluation Project survey	Intervention: the LIFT+ program - consists of 8 one hour workshops that focus on the negative effects of smoking, the benefits of eating fruits and vegetables, incorporated goal setting, and activities for the students to complete with their families. Once the students finished the LIFT+ program they would then teach a shortened version of the program to the younger kids.	Fruit and vegetable consumption was significantly higher for the intervention group. At one year follow up, fruit consumption was only marginally higher and vegetable consumption was only significantly higher for white children in the intervention group compared to the control group. Intervention students could correctly identify the recommendation for fruits and vegetables per day after the intervention but this decreased at one-year follow up.	Self-reported

**Table 2:** General characteristics of included studies^a^

	**Randomized controlled trial**	**Non-randomized controlled Trial**
Total number of studies	5	9
Participants	5738	6769
Average intervention length	19.8 months	11.8 months
Multi-component interventions	4	9
Intervention components		
Teacher involvement	1	5

^a^References 25-38.

**Table 3:** Summary of positive and non-significant findings for increasing fruit and vegetable consumption^a^

	**Positive finding**	**Non-significant finding**
Multi-component interventions (n=13)	8	5
Intervention components		
Teacher involvement (n=6)	1	5
Food service staff involvement (n=2)	2	0
Parent/family involvement (n=7)	3	4
Garden-based (n=2)	1	1
Theory-based (n=9)	5	4
Antecedent for increasing consumption e.g. knowledge, preference, self-efficacy, attitudes? (n=7)	2	5
Study design		
Randomization (n=5)	3	2
Non-randomization (n=9)	5	4

^a^References 25-38.

### Inclusion and exclusion criteria

To be included, studies were required to be full-text original research published in academic peer-reviewed journal, written in English language, administered in the United States, and conducted among a population-based sample of children in Kindergarten through eighth grade. Each study was also required to examine the population’s fruit and vegetable consumption. Once articles were selected based on the inclusion criteria, they were then evaluated based on the exclusion criteria. Articles were excluded if the study did not include a control group, was neither experimental nor quasi-experimental, did not have qualitative data, did not include a school nutrition education component, and was not a school-based intervention. Moreover, review articles, conference abstracts, commentaries, guest editorials, letters to editor, and brief reports were excluded from the study.

### Data extraction 

Microsoft Excel, 2013 was used to build a data extraction table. Two reviewers (CRA and TAS) independently extracted the data, and then the extracted data was cross-checked for the accuracy. Any data discrepancies were resolved through mutual consensus between the reviewers. This systematic review was conducted to collect and summarize the research on school-based nutrition interventions measuring fruit and vegetable consumption/intake. Since the goal was to explore the most recent interventions used to increase fruit and vegetable consumption in primary-school aged children, we did not conduct further meta-analysis.

## Results

A total of 14 articles, published between 2006 and 2014, were included, on effects of school-based nutrition interventions effect on students’ fruit and vegetable consumption. The studies included in this systematic review were summarized based on design, sample, methods, intervention, and key results (see Table 1). Figure 1 presents the literature search strategy.

A total of 14 publications met the inclusion criteria. Of those, eight significantly increased children’s consumption of fruit, vegetable or both, while the other six showed no significant increase. Since one of the inclusion criteria was to have a control group, all studies were required to have this feature. Of the 14 studies, five used randomization and the other nine used non-random or convenience-style sampling (Table 2). The five randomized-control trials yielded three positive outcomes and two non-significant outcomes for increasing fruit and vegetable consumption (Table 2).

Even though the studies ranged from moderate to good in their quality ratings, they were all observational and many used self-reported outcomes, which is often biased. Additionally, secular trends could not be ruled out; fruit and vegetable consumption is a very popular trend both in educational settings and among popular culture. Another finding from our review was that there was absolutely no correlation between higher fruit and vegetable consumption and intervention length.

The average length of an intervention in our review was 19.8 months for randomized studies and 11.8 months for non-randomized studies (Table 2). Moreover, six of the 14 studies in this review had a sample size greater than 500, with four studies with a sample size of less than 200. Overall, the quality of the evidence is good and given the high level of stringency for inclusion in our study, our review deepens the understanding of school-based nutrition interventions.

## Discussion

After reviewing the included studies we found the most commonly used intervention was parent or family involvement. Of those seven, three studies^27,36,38^ reported significant increase in fruit and vegetable consumption, while four did not.^25,30,31,35^ Another common component was teacher involvement with (6 out of 14). Of those six only one reported a significant increase in fruit and/or vegetable consumption,^36^ while five did not (Table 3).^30-32,34,35^ One possible explanation for both findings comes from a recent study conducted in Serbia, by Šumonja and Novaković. These researchers found a negative relationship between students consuming less fruit and vegetables and parents and teachers “telling” them to consume more. However, they also found that the “*perceived norm of parental eating behavior is a significant influence on children’s intake of fruits, vegetables*….”^39^

We were able to determine the more effective interventions included a nutrition education component with three positive findings^26,28,33^ and one non-significant finding.^29^ Additionally, food service components were found to positively affect fruit and/or vegetable consumption without non-significant findings.^26,33^ Before we can promote these interventions more studies should be conducted.

There has been great enthusiasm for garden-led interventions and while our selection only captured two studies that included gardens, both of those saw non-significant outcomes for fruit and/or vegetable consumption (Table 3).^28,29^ Even though our review did not specifically target school gardens, we assumed the broad inclusion of all school-based interventions that measured fruit and vegetable consumption would have yielded a larger amount of garden-based studies. In 2009, a systematic review was conducted on that specific topic and included 11 studies.^23^ Of those 11, four did not have a control group, and seven studies did not measure actual fruit and vegetable consumption, but some antecedent for fruit and vegetable consumption. Given those results, it is surprising that the authors concluded “*garden-based nutrition intervention programs may have the potential to promote increased fruit and vegetable in-take among younger children*” (p. 273).^23^ Notice the authors did not claim that garden-based interventions have the potential to increase student consumption of fruit and vegetables, but that school gardens have the potential to promote a step in the right direction. Interestingly, in 2014, the USDA has invested five million dollars in farm-to-school programs^40^ and according to a census conducted by the USDA, 2401 schools have a garden out of 40 328 who took the survey.^41^ The trend promoting school gardens is an interesting one and we would like to see more research guiding these public investments.

Another area that has garnered much attention by reviewers is whether single or multiple components in the intervention.^18-23^ Most reviews tend to stop their examination at whether it was multi-component or not.^18-23^ In their 2012 review, Evans et al, also declared that multi-component interventions led to “larger improvements” in fruit and vegetable consumption but tempered their claim by saying that multi-component interventions could be “difficult to replicate.”^19^ Of the 14 studies in our review, 13 were multi-component with eight registering a positive finding^26-29,33,36-38^ and five reported non-significant findings (Table 1).^30-32,34,35^ Our findings are in agreement with Evans and colleagues that reported multi-component interventions were effective, but there were still a lot of nonsignificant findings which leads one to question whether multi-component interventions are a meaningful category, and not simply a way of maintaining an expert status without providing substantial contributions of knowledge to the school-based nutrition interventions.

### Summary of theory-based interventions 

The studies reviewed also utilized theoretical models to develop their interventions. Nine of the 14 articles employed a theory, and of those nine, only five were positive,^27,36-38^ while the other four had not significantly increased fruit, vegetable or both fruit and vegetables combined.^29-32,35^ Furthermore, of the remaining five studies that did not employ a theory, there were three positive findings,^26,28,33^ and two non-significant ones (Table 3).^24,34^ This finding is in contrast with the findings of Rush and Knowlden who reviewed 11 articles, four of which did not have a control group and concluded that despite limitations, they recommended working on self-efficacy for school and community-based interventions.^18^ When breaking the theory-based findings down by whether they were randomized or not, one of the nine employed randomization that yielded a positive outcome (Table 3).^38^ Clearly, the results of this review indicate that it is difficult to draw an association between the employment of a theory in an intervention and any increase in consumption of fruit, vegetable or both by the student. This finding corresponds with Baranowski et al study, which questioned whether health behavioral change models are effective in controlling the obesity epidemic.^42^

### Summary of measuring consumption vs. antecedents

In this section of the review, we created a general category of “antecedent” to actual fruit and vegetable consumption. This category included any variable that measured knowledge, preference, attitude, or self-efficacy the purpose of doing this was to point out that some reviews, and the studies, have used antecedents as a proxy for increased consumption of fruit and vegetables.^18,23^ If a study found a positive association for a particular intervention component with an antecedent the authors would claim that intervention was successful in improving fruit and vegetable consumption or sometimes the word “promoting” fruit and vegetable consumption was used. Consequently, there seems to be some incongruence between the enthusiasm for school-based nutrition interventions and the actual evidence for increasing fruit and vegetable consumption. Our review examined 14 studies, seven of which measured consumption along with antecedents. Of those seven, five found that antecedents did not correlate with a higher consumption of fruit consumption alone, vegetable consumption alone, or combined (Table 3).^27,30-32,34^ Only two studies reported a positive outcome for fruit and/or vegetable consumption and one antecedent.^29,38^

### Recommendation for future research 

The results of our review indicate that little is known as to what constitutes an effective school-based nutrition education program. Since large amounts of public money used to fund these types of interventions, more research should be done to attempt to ascertain the most effective ingredients of a school-based nutrition intervention. Our review helps to rule out several components that are not associated with positive outcomes: Parental or family involvement, teacher involvement, attempting increase fruit and vegetable consumption by increasing antecedents of fruit and vegetable consumption, and employing a behavior change theory. These components should be avoided in future research. These recommendations might seem overly stringent but the above components had more non-significant findings than positive in our review, and when we include the current shortage of research funding along with time constraints, it seems prudent to put that money and time to better use. Our review’s modest positive outcomes came from the components that included working with the food service staff and using standard nutrition education. We would recommend including one or both of these components in a school-based-nutrition intervention. We also recommend that researchers focus on increasing both fruit and vegetable consumption and measure them separately and jointly. This would allow for more specific observations on fruit and vegetable consumption. Again, it is essential that more research further examine what constitutes a successful intervention.

## Limitations

Our systematic review has some following limitations that warrant consideration. Inclusion of articles only written in English-language could have led to an introduction of bias, but it was reported that language bias has minimum impact on the conclusions of systematic reviews.^43^ Second, because only PubMed was used for the literature search, it is plausible that some studies relevant to this review have not been captured. PubMed was chosen due to its extensive library. It consists of over 24 million references and includes MEDLINE database. Moreover, we used a wide range of keywords for the search process to gather greater number of potential studies. However, we believe that searches in multiple electronic databases would have added more comprehensive insight and given a more complete picture of the school-based nutrition intervention landscape. Third, as a majority of the studies included were self-report survey-based, this systematic review is subject to recall bias. More studies should consider direct observation of participants’ actual behavior. Also, reports of teachers and parents could be an effective strategy to provide more complete picture. Finally, all the studies were conducted in the United States; therefore, it might be hard to generalize these results to other countries. Despite limitations, this review certainly highlights and provides comprehensive understanding of intervention components and effectiveness of research in this area.

## Conclusion

The main findings of this review were that, even with a high inclusion criteria placed on research design quality, there was no conclusive intervention type or component that could be associated with increasing fruit and vegetable consumption in public school students in the United States. Given that the criteria for selection were high, lack of consistency between interventions and positive outcomes was surprising. Additionally, this paper raises concerns about the use of a category called “multicomponent.” It is imperative that more research be conducted to identify the most effective intervention components.

## Ethical approval

We have observed appropriate ethical guidelines and legislation in conducting the study. Since this was a systematic review, approval was not required by the institutional review board.

## Competing interests

The authors do not have any financial or personal conflict of interest related to this of this systematic review.
